# Impact of Scannable Healing Abutment Type on the Accuracy of Implant Impression

**DOI:** 10.1155/bmri/1061741

**Published:** 2026-02-25

**Authors:** Han Na Lee, Yeon-Kyung Park, Ji Suk Shim, Jeong-Yol Lee

**Affiliations:** ^1^ Department of Dentistry, Korea University Guro Hospital, Seoul, Republic of Korea, kumc.or.kr

## Abstract

**Objectives:**

The objectives of this study are to determine whether scannable healing abutment (SHA) geometry affects implant‐impression accuracy compared with conventional scan bodies (SBs) and to assess the effect of a detachable cap on SHA accuracy.

**Methods:**

Three partially edentulous mandibular models were fabricated, each with two implants at the right second premolar and first molar, corresponding to three implant systems: IS‐III active (Neobiotech), TS‐III (Osstem), and Bright tissue level (Dentium). For each system, scans were obtained with SBs and SHAs; in the Bright system, a detachable‐cap SHA (SHAC‐B) was additionally tested. The seven groups were SB‐I, SB‐T, SB‐B, SHA‐I, SHA‐T, SHA‐B, and SHAC‐B (*n* = 10 scans per group). Reference datasets were acquired with a laboratory scanner (inEos X5), and intraoral scans were obtained with an intraoral scanner (Primescan, Dentsply Sirona). Implants were reconstructed in exocad Dental CAD 2.2 and analyzed in Geomagic Control X after best fit alignment to adjacent teeth. Outcomes were 3D linear and implant angular deviations. Wilcoxon signed‐rank tests compared SBs with SHAs within each system and SHA‐B with SHAC‐B; differences among SHA‐I, SHA‐T, and SHA‐B were assessed with Kruskal–Wallis tests and Bonferroni‐adjusted pairwise comparisons (*α* = 0.05).

**Results:**

3D linear deviations were < 70 *μ*m for all groups except SHAC‐B. For 3D linear deviation, *p* values (second premolar, first molar) were 0.017, 0.139 (SB‐I vs. SHA‐I); 0.005, 0.013 (SB‐T vs. SHA‐T); and 0.241, 0.169 (SB‐B vs. SHA‐B). Corresponding angular *p* values were 0.005, 0.005; 0.005, 0.005; and 0.074, 0.017, respectively. In the Bright system, adding a cap (SHA‐B vs. SHAC‐B) reduced accuracy (linear 0.009, 0.037; angular 0.005, 0.005). Among SHA groups, differences occurred only at the second premolar, where SHA‐B differed from SHA‐I and SHA‐T; no differences were observed at the first molar.

**Conclusions:**

SHA geometry influenced implant‐impression accuracy, yet deviations were generally within clinically acceptable ranges. Cap application reduced accuracy, highlighting the need to optimize cap design and connection.

## 1. Introduction

Accurately transferring the three‐dimensional location of the implants relative to other intraoral tissue structures is essential for achieving a precisely fitting prosthesis [1–3]. Various techniques have been proposed to enhance transfer accuracy, including conventional impression methods and emerging digital workflows [4–6]. If the impression is inaccurate, it may result in an ill‐fitting prosthesis, thereby increasing the risk of mechanical and/or biological complications [1, 6, 7]. While achieving a completely passive fit may not be feasible, minimizing misfit to avoid potential complications remains a widely accepted goal in implant prosthodontics [8–10].

The remarkable development of computer‐aided design and computer‐aided manufacturing (CAD‐CAM) technology brought digitalization to implant dentistry [11–14]. Digital impression techniques provide clinicians an alternative to conventional impression methods by using scan bodies (SBs) and intraoral scanners [4, 15]. SBs are commonly fabricated from polyetheretherketone (PEEK), titanium or aluminum alloys, and high‐performance resins. Material properties such as reflectivity, translucency, color, surface texture, and stiffness influence how the scanner captures light and edges. These properties can change trueness and precision [11, 16]. Manufacturing tolerances and the fit at the implant–abutment interface also matter. These factors can compromise library alignment and the accuracy of the transferred implant position [11, 15]. Digital impressions simplify the impression process and reduce chair time and patient discomfort [17–19]. In addition, they improve communication between clinicians and dental laboratories, enhancing overall workflow efficiency [20, 21]. Several studies have demonstrated that the accuracy of digital implant impressions using SBs and intraoral scanner is comparable to that of conventional implant impressions [16, 22, 23]. Furthermore, subsequent research has validated the clinical applicability of digital impressions in both partially and completely edentulous cases, reporting acceptable levels of trueness and precision in terms of three‐dimensional deviation and angular accuracy [24–27]. For these reasons, digital implant impressions are now widely adopted in clinical practice.

However, the use of SBs still has limitations, such as the need to remove the healing abutment, which may cause patient discomfort and disrupt peri‐implant soft tissue [14, 19, 27]. Repeated removal and reconnection of abutments may lead to disruption of the peri‐implant soft tissue, potentially inducing apical migration of the junctional epithelium [14, 18]. Clinical evidence further suggests that such repeated manipulation can compromise soft‐tissue stability and integrity around implants [28, 29]. To address these challenges, scannable healing abutments (SHAs) have been introduced as a hybrid component that enables direct intraoral scanning while maintaining the healing function, thereby performing the roles of both the healing abutment and the SB [28–30]. SHAs are manufactured from the same material families commonly used for SBs and conventional healing abutments, and their material and surface characteristics influence optical readability, seating repeatability, and library‐alignment fidelity [11, 15]. This term encompasses designs reported in the literature, including SHAs with surface‐encoded geometry and healing‐abutment scan‐peg mechanisms [29, 31–33]. Accordingly, SHA‐based workflows obviate repeated abutment removal during impression procedures, preserving soft‐tissue architecture and improving patient comfort [14, 28, 29]. Furthermore, the use of SHAs can enhance procedural efficiency and reduce overall treatment costs by minimizing the number of required components [11, 30]. Consistent with these benefits, a randomized clinical trial reported that an SHA‐based workflow shortened data acquisition time while maintaining clinical contact quality and occlusal performance relative to a SB approach [31]. A clinical study evaluating a newly designed SHA with surface‐encoded geometry reported high prosthetic precision with stable hard and soft‐tissue outcomes and a low incidence of complications, supporting the feasibility of a simplified digital workflow [32]. Complementing these findings, an in vitro investigation showed that registration accuracy between soft‐tissue and implant‐position scans depends on the scanning system and that an SHA with surface‐encoded geometry enabled automatic alignment with accuracy comparable to conventional approaches [33]. A prior study reported that SHA designs with surface‐encoded geometry can simplify clinical steps and support cost‐effective implant prosthodontics [29]. Despite these advancements, the determinants of impression accuracy with SHAs, particularly abutment geometry and height as well as alignment‐related variables, remain insufficiently characterized.

Therefore, the purpose of this in vitro study was to evaluate the impact of the shape and height of SHAs on the accuracy of implant impressions. The first null hypothesis was that the shape of the SHA would not influence implant impression accuracy. The second null hypothesis was that increasing SHA height with a detachable cap would not influence implant impression accuracy.

## 2. Materials and Methods

SBs and SHAs from three different implant systems were compared and evaluated in this study (Table 1). A partially edentulous mandibular dentiform missing the right second premolar and first molar was scanned, and three identical models were fabricated using a 3D printer (Max X; Asiga, Sydney, Australia) and printing resin (JAMG HE Standard 3D Printer UV Resin, Shenzhen Yongchanghe Technology Co. Ltd., China). The printing was conducted with a layer thickness of 50 *μ*m. Key printing parameters were set as follows: normal layer exposure time 3.0 s and bottom layer exposure time 30 s. After printing, the models were thoroughly cleaned in 99% isopropyl alcohol for 2 min using an ultrasonic cleaner to remove uncured resin residue. The models were then dried completely with compressed air and post‐cured for 10 min.

**Table 1:** List of components used.

Implant	Scan body	Scannable healing abutment	Manufacturer
IS‐III active (4 × 10 mm)	Intraoral scan body (GMS‐DERNS; height: 7.5 mm)	Healing scan body IS R (KSR45H5H; height: 5 mm)	Neobiotech Co. (scan body); Geo GMS (scannable healing abutment), Seoul, Korea
TS‐III (4 × 10 mm)	Scan body regular (TSSBOS; height: 10 mm)	Osstem scannable healing abutment (TSSHA445R; height: 5 mm)	Osstem Implant Co., Seoul, Korea
Bright tissue level (4 × 11 mm)	Bright screw metal link (BSMI4030H; height: 8.5 mm)	Bright scan comfort cap (BISC3848H; height: 5 mm)	Dentium Co., Seoul, Korea

Fixtures from the three implant systems were placed 1 mm below the model surface, parallel at the right second premolar and first molar positions in each printed mandibular model: IS‐III active (Neobiotech Co., Seoul, Korea), TS‐III (Osstem Implant Co., Seoul, Korea), and Bright tissue level (Dentium Co., Seoul, Korea).

For each system, scans were obtained with SB and with SHA. In the Bright system, a detachable‐cap SHA (SHAC) was additionally tested. Suffixes ‐I, ‐T, and ‐B denote the IS‐III active, TS‐III, and Bright tissue level systems, respectively. The SB data were categorized as SB‐I (IS‐III active), SB‐T (TS‐III), and SB‐B (Bright tissue level) (Table 2).

**Table 2:** Experimental design: intraoral scanning of different implant systems.

Groups	Implant system and scanning component	Sample size (*n*)
SB‐I	IS‐III active implant with connected scan body	10
SB‐T	TS‐III implant with connected scan body	10
SB‐B	Bright tissue level implant with connected scan body	10
SHA‐I	IS‐III active implant with scannable healing abutment	10
SHA‐T	TS‐III implant with scannable healing abutment	10
SHA‐B	Bright tissue level implant with scannable healing abutment	10
SHAC‐B	Bright tissue level implant with capped scannable healing abutment	10

*Note:* Laboratory scan data were used as the reference for deviation calculations in all groups.

After SBs corresponding to each implant system (SB‐I for IS‐III active, SB‐T for TS‐III, and SB‐B for Bright tissue level) were connected, a single scan was performed using a laboratory scanner (inEos X5, Dentsply Sirona, Bensheim, Germany) to obtain the standard tessellation language (STL) dataset for each group. Subsequently, 10 consecutive full‐arch scans for each group were acquired for each implant system by a single operator using an intraoral scanner (PRIMESCAN, Dentsply Sirona, Bensheim, Germany) to generate the experimental STL data. Scans followed the manufacturer‐recommended sequence. The scanning was initiated on the occlusal surface of the right second molar, where the scanner tip was immediately tilted approximately 60° in a lingual direction and moved along the dental arch to the opposite left second molar. The scanner was then guided over the occlusal/incisal surfaces from the left second molar across the entire dental arch back to the right side. To complete the main sweep, the scanner was tilted approximately 60° in a buccal direction and moved along the entire buccal aspect of the dental arch.

Subsequently, the SBs were removed, and SHAs were attached to each implant system (Figure 1). Both laboratory and intraoral scanning were repeated to obtain SHA‐I (IS‐III active), SHA‐T (TS‐III), and SHA‐B (Bright tissue level) datasets. For the SHA‐B group, a cap was manually attached to form SHAC‐B, aligning it with the SHA top geometry. A hexagon‐shaped SHA was used, with all indexing notches oriented buccally. Both SBs and SHAs were connected to the implants using a torque ratchet of 10 Ncm.

Figure 1Scannable healing abutment (SHA) systems evaluated in this study. (a) Healing scan body IS R (Neobiotech Co., Seoul, Korea). (b) Osstem scannable healing abutment (Osstem Implant Co., Seoul, Korea). (c) Bright scannable healing abutment (Dentium Co., Seoul, Korea). (d) Bright scannable healing abutment with detachable cap (Dentium Co., Seoul, Korea).

**Figure:**
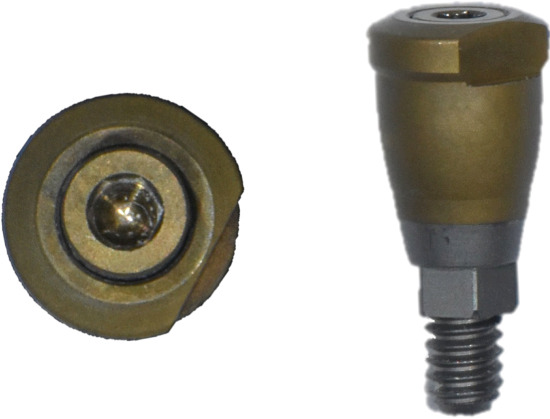
(a)

**Figure:**
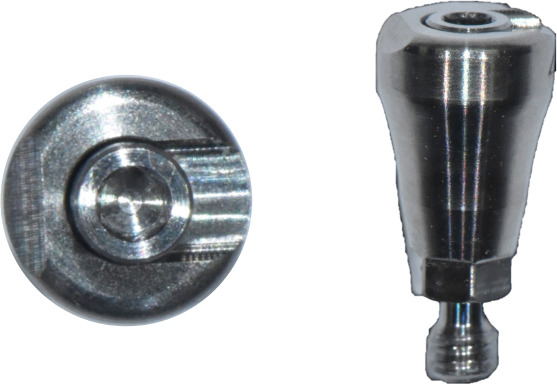
(b)

**Figure:**
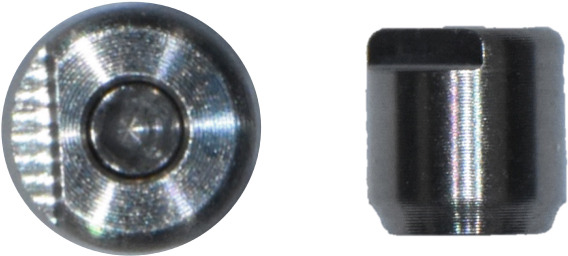
(c)

**Figure:**
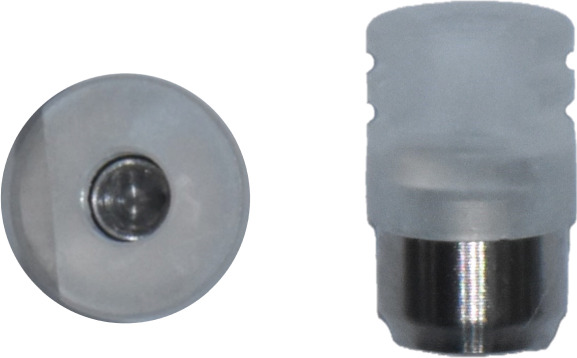
(d)

The implants were positioned from the implant library with a CAD‐CAM software program (exocad Dental CAD 2.2 Valletta; exocad GmbH). The implant positions and long axes were defined using the manufacturer‐specific library of each implant system, and the completed designs were exported in STL data. A 3D analysis program (Geomagic Control X; 3D Systems) was used to analyze the exported STL dataset. The reference STL obtained from the laboratory scanner was defined as the reference dataset, and each intraoral scan was imported as the test dataset. Both datasets were aligned by selecting three corresponding anatomical landmarks on the adjacent teeth, followed by a best fit alignment limited to those regions.

The accuracy of each group was evaluated by calculating both 3D linear and angular deviation relative to the reference data. A reference plane was established from three anatomical points on the adjacent teeth surrounding the edentulous area of the cast (Figure 2). The plane served as the local coordinate reference for angular measurements. The long axis of each implant was reconstructed from the CAD library, and the angle between the implant axis and the reference plane was measured using the Angular Dimension function in Geomagic Control X. The absolute difference between the reference and test angles was defined as the implant angular deviation (Figure 3).

Figure 2Definition of the reference plane and coordinate system. (a) Three reference points were identified on the mandibular model: (1) the mesiobuccal cusp tip of the right second molar (point A), (2) the mesiolingual cusp tip of the right second molar (point B), and (3) the buccal cusp tip of the right first premolar (point C). A reference plane (blue surface) was defined by the three points. (b) The *x*‐ and *y*‐axes were aligned on the reference plane, and the midpoint between points A and C designated as the coordinates origin (yellow dot). (c) The *z*‐axis was established as a line perpendicular to the reference plane and passing through the origin.

**Figure:**
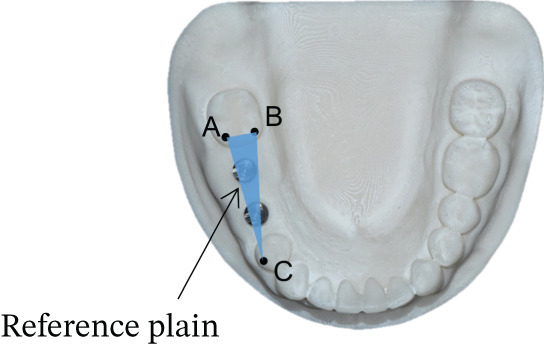
(a)

**Figure:**
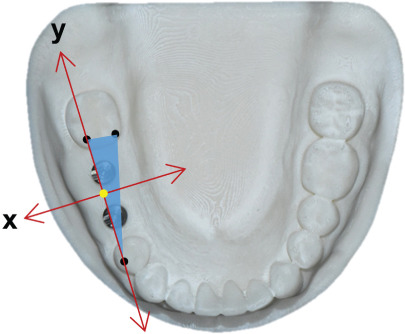
(b)

**Figure:**
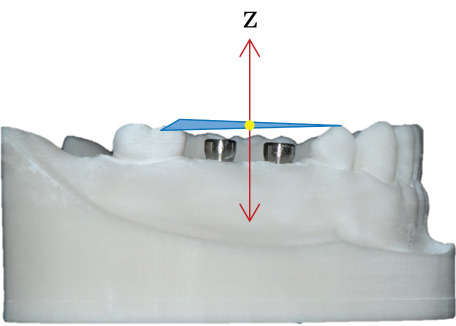
(c)

Figure 3Establishment of the reference plane and measurement of implant angular deviation. (a) A reference plane (blue surface) was created by selecting three anatomical reference points on the adjacent teeth surrounding the edentulous area. (b) The implant axis (green lines) was defined for each implant, and the angle between the implant axis and the reference plane (blue surface) was measured to determine the angular deviation.

**Figure:**
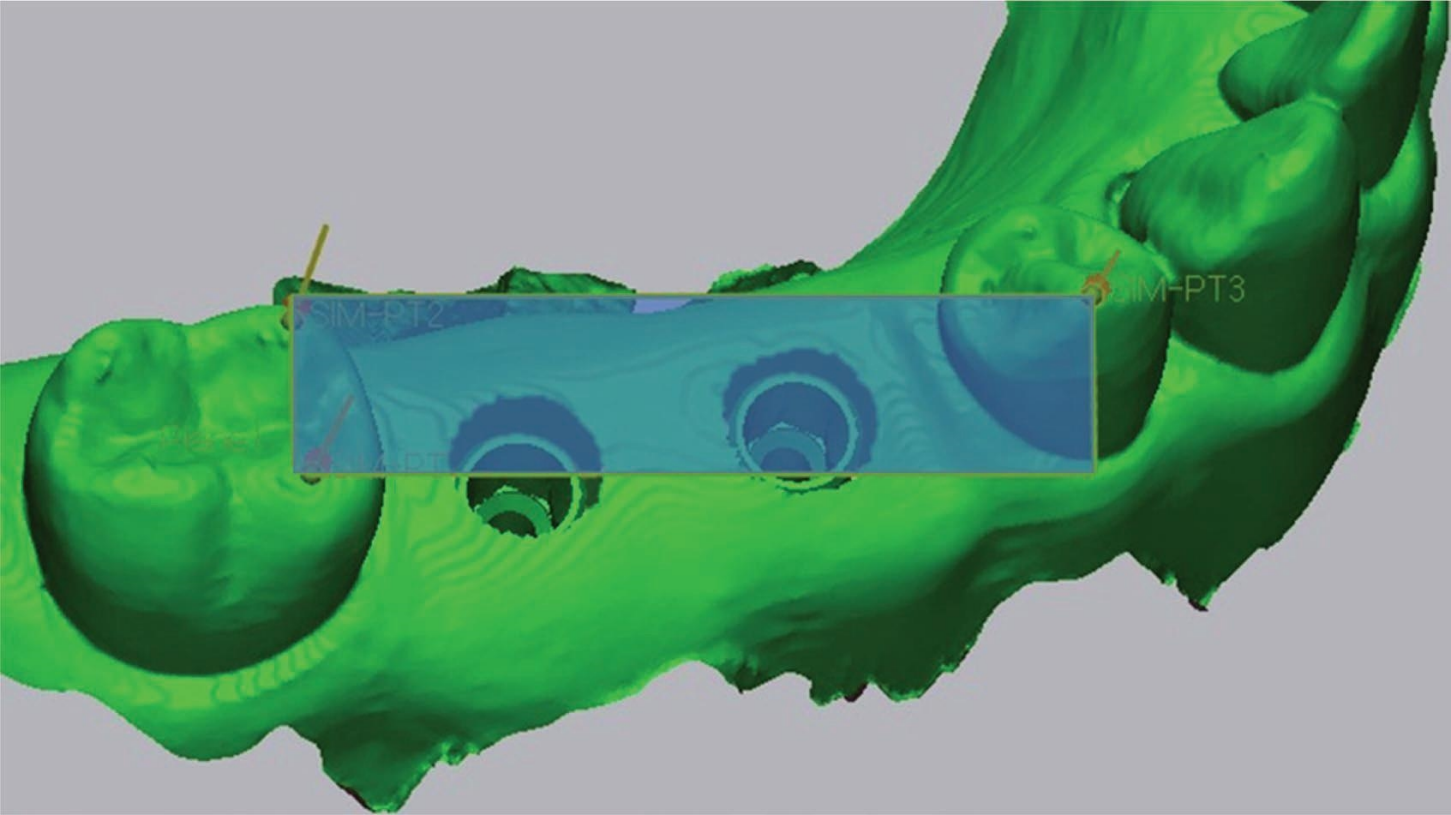
(a)

**Figure:**
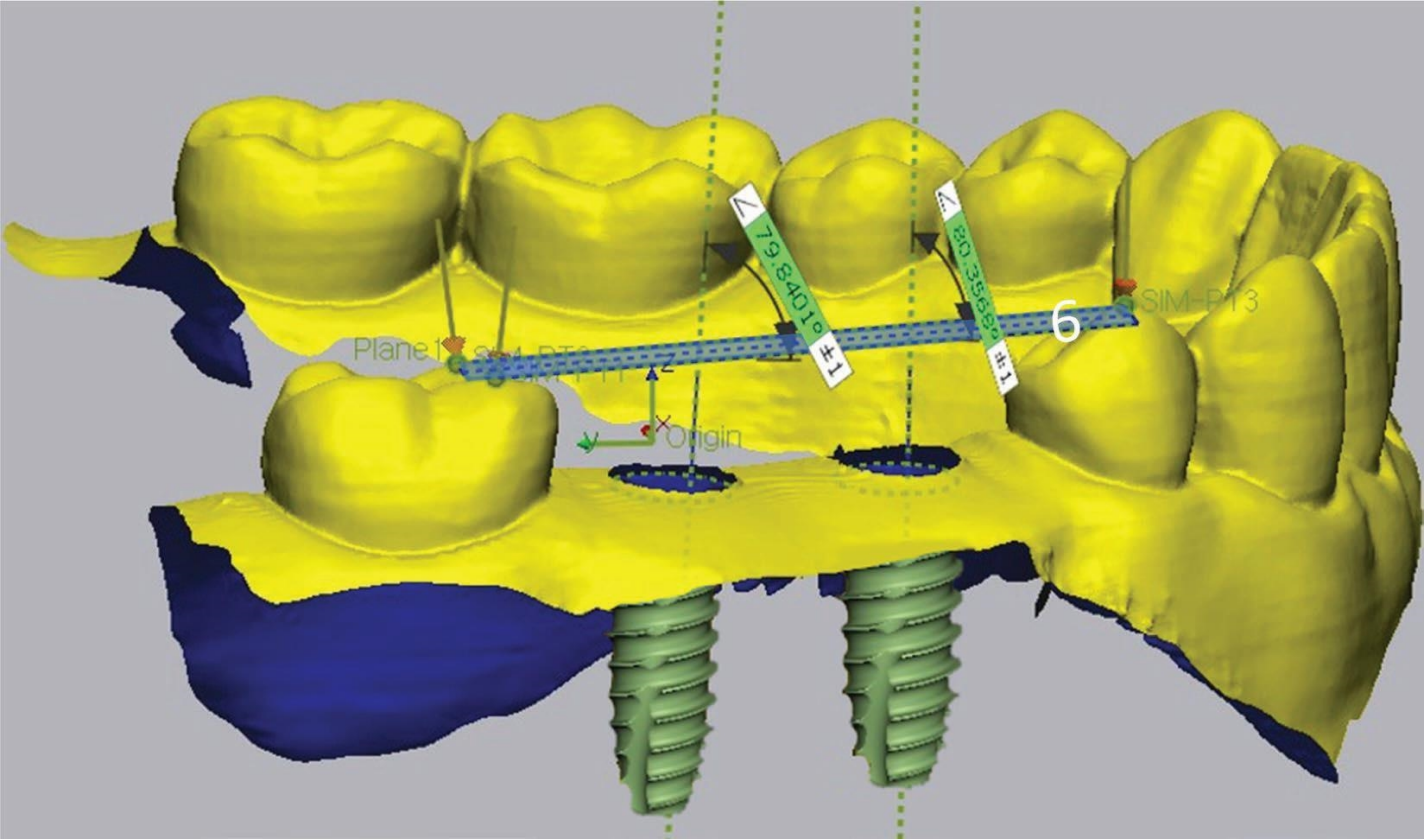
(b)

For the linear deviation analysis, the center of the implant platform was determined using the Circle Center function in Geomagic Control X, and the coordinates (*X*, *Y*, *Z*) were extracted for both the reference and test datasets (Figure 4). The linear deviations (*Δ* *X*, *Δ* *Y*, *Δ* *Z*) were calculated for each axis, and the total 3D linear deviation was computed using the following formula: $\text{Total}\;\text{deviation}=\sqrt{{\left(\Delta X\right)}^{2}+{\left(\Delta Y\right)}^{2}+{\left(\Delta Z\right)}^{2}.}$

Figure 4Measurement of the center point at the implant fixture platform. (a) A circular plane (orange ellipse) was created at the implant platform by selecting three reference points (sky blue dots) along the implant shoulder. (b) The center point of the implant fixture platform (yellow dot) was defined as the intersection between the implant axis (red solid line) and the fitted circular plane (orange ellipse).

**Figure:**
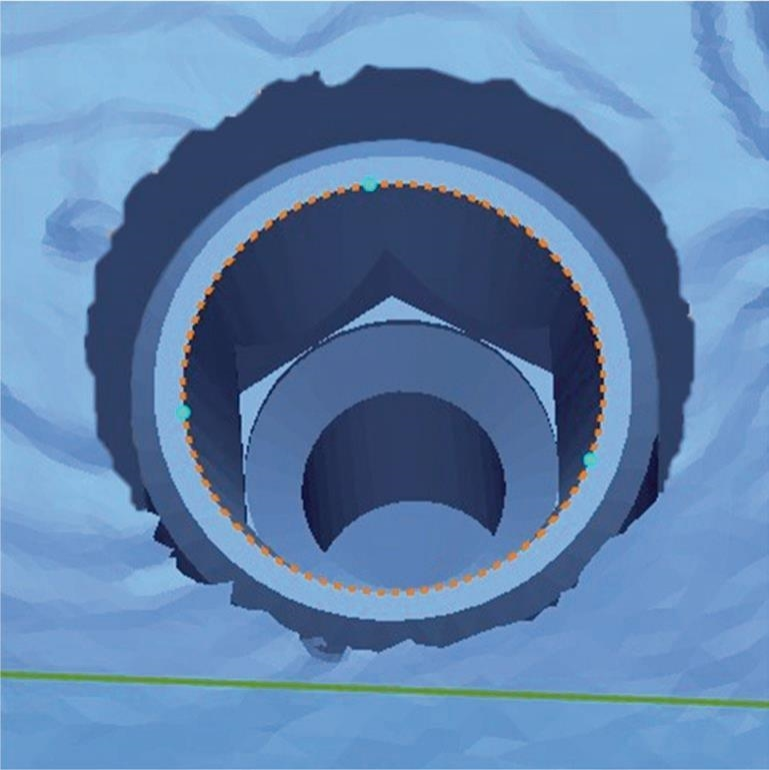
(a)

**Figure:**
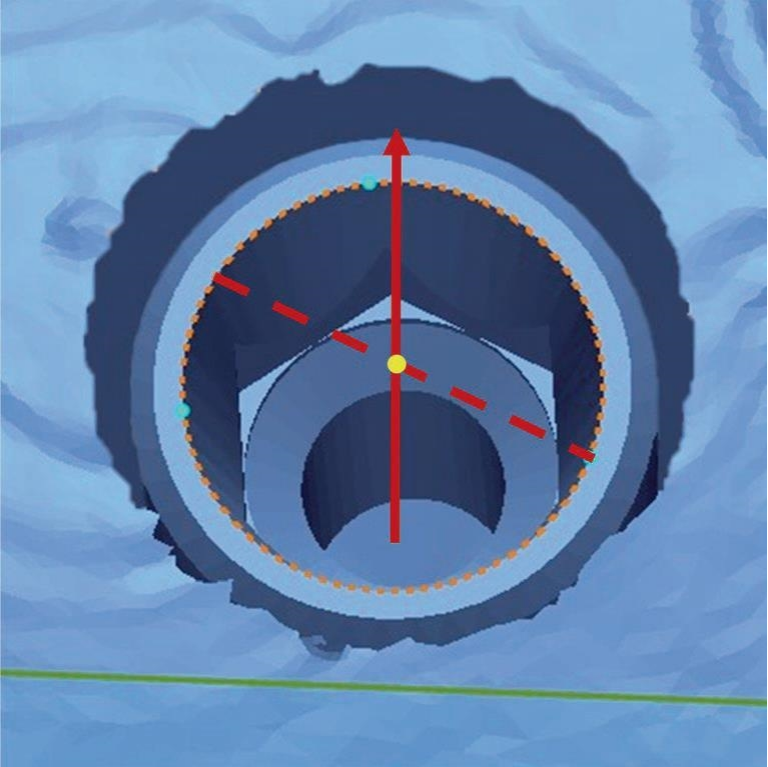
(b)

Statistical analysis was performed using SPSS Version 21.0 (IBM Corp., Armonk, New York, United States). The normality of the data distribution was assessed using the Shapiro–Wilk test. As the data did not meet the assumption of normality, nonparametric tests were used. For paired comparisons between SB and the corresponding SHA, as well as between SHA‐B and SHAC‐B, the Wilcoxon signed‐rank test was applied. Differences among the three SHA groups (SHA‐I, SHA‐T, and SHA‐B) were evaluated using the Kruskal–Wallis test, followed, when appropriate, by Bonferroni‐adjusted pairwise comparisons. For all statistical tests, *p* < 0.05 was considered statistically significant.

## 3. Results

The mean 3D linear deviations were less than 70 *μ*m in all groups except for SHAC‐B (second premolar: 187.6 ± 220.1 *μ*m, first molar: 271.5 ± 299.8 *μ*m), which was significantly greater than the other groups (Table 3).

**Table 3:** Mean ± standard deviation of 3D linear (*μ*m) and implant angular (°) deviations for each group.

Groups	3D linear deviations (*μ*m)		3D implant angular deviations (°)	
	Second premolar	First molar	Second premolar	First molar
SB‐I	37.0 ± 22.2	45.6 ± 23.6	0.061 ± 0.097	0.642 ± 0.064
SB‐T	38.3 ± 7.1	46.1 ± 13.2	0.124 ± 0.045	0.166 ± 0.035
SB‐B	43.3 ± 7.8	63.0 ± 14.7	0.207 ± 0.139	0.146 ± 0.104
SHA‐I	60.7 ± 7.9	59.6 ± 10.9	0.284 ± 0.105	0.113 ± 0.08
SHA‐T	58.6 ± 9.6	63.3 ± 13.3	0.234 ± 0.042	0.045 ± 0.046
SHA‐B	45.7 ± 34.0	56.8 ± 23.7	0.112 ± 0.056	0.048 ± 0.05
SHAC‐B	187.6 ± 220.1	271.5 ± 299.8	1.333 ± 1.413	1.606 ± 1.136

*Note:* Values are expressed as mean ± standard deviation.

Abbreviations: B, Bright tissue level; I, IS‐III; SB, scan body; SHA, scannable healing abutment; SHAC, scannable healing abutment with cap; T, TS‐III.

When comparing the SB and SHA within each implant system, a statistically significant difference was observed in the 3D linear deviation between groups (*p* < 0.05), except for the first molar in IS‐III active (*p* = 0.139) and both the second premolar (*p* = 0.241) and first molar (*p* = 0.169) in Bright tissue level. For 3D implant angular deviation, significant differences were observed in all comparisons (*p* < 0.05), except for the second premolar (*p* = 0.074) in the Bright tissue level system. Statistically significant differences were found between the SHA‐B and SHAC‐B groups in both 3D linear and implant angular deviations (*p* < 0.05) (Table 4).

**Table 4:** Significance levels for 3D linear and implant angular deviations across SB and SHA groups.

Groups	3D linear deviations		3D implant angular deviations	
	Second premolar	First molar	Second premolar	First molar
SB‐I vs. SHA‐I	0.017	0.139	0.005	0.005
SB‐T vs. SHA‐T	0.005	0.013	0.005	0.005
SB‐B vs. SHA‐B	0.241	0.169	0.074	0.017
SHA‐B vs. SHAC‐B	0.009	0.037	0.005	0.005

*Note:* Differences were considered statistically significant at *p* < 0.05.

Abbreviations: B, Bright tissue level; I, IS‐III; SB, scan body; SHA, scannable healing abutment; SHAC, scannable healing abutment with cap; T, TS‐III.

Among SHA groups, at the second premolar both 3D linear deviation and implant angular deviations differed significantly (*p* < 0.05): SHA‐B exhibited significantly lower deviations than SHA‐I and SHA‐T, whereas SHA‐I and SHA‐T did not differ. At the first molar, no intergroup differences were detected (Figure 5).

Figure 5Comparison among SHA geometries at the second premolar and first molar: (a) 3D linear deviation (*μ*m); (b) 3D implant angular deviation (°). Box plots display SHA‐I, SHA‐T, and SHA‐B (*n* = 10 per group) analyzed within each site (second premolar, first molar). Boxes indicate the interquartile range with the median; whiskers represent the data range. Within each site, groups sharing the same superscript letter do not differ significantly, whereas different letters indicate significant differences (Kruskal–Wallis, followed by Bonferroni‐adjusted pairwise comparisons, *α* = 0.05). Abbreviations: SB, scan body; SHA, scannable healing abutment; SHAC, scannable healing abutment with cap; I, IS‐III; T, TS‐III; B, Bright tissue level.

**Figure:**
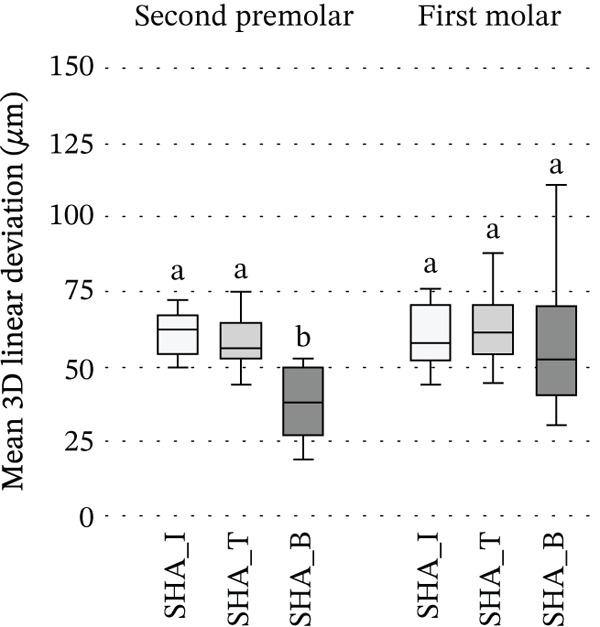
(a)

**Figure:**
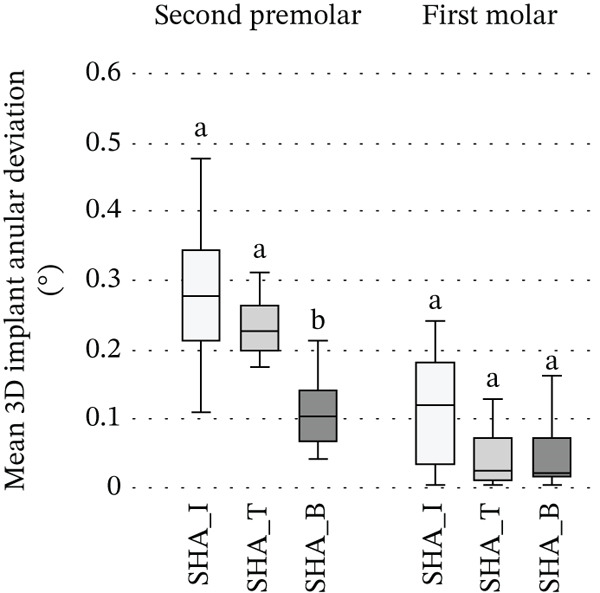
(b)

## 4. Discussion

Based on the results of this study, significant differences in accuracy were observed among the three SHAs with different shapes, leading to the rejection of the first null hypothesis. Additionally, significant differences in accuracy were found between the SHA and the capped SHA, resulting in the rejection of the second null hypothesis.

To the authors’ knowledge, no studies have previously focused on evaluating the impact of SHA shapes on scan accuracy. However, geometrical characteristics of SBs, including their shape, surface configuration, and indexing design, play a critical role in the acquisition of accurate three‐dimensional data during intraoral scanning [3, 5, 11]. This is supported by findings from Mizumoto et al. [27] and Batak et al. [30], who reported that variations in SB geometry significantly influence the trueness of digital implant impressions. Scanner tracking and digital data processing can be affected by SB material properties, surface reflectivity, and structural geometry [3, 15, 26]. These factors have been demonstrated to impact the accuracy of digital impressions, particularly in terms of spatial resolution and angular accuracy [27, 28, 30]. In this study, comparisons between each implant system’s SB groups and SHA groups demonstrated statistically significant differences in both 3D linear and 3D implant angular deviations.

The intraoral scanner used in this study (Primescan, Dentsply Sirona, Bensheim, Germany) operates with Dynamic Depth Scan (DDS) technology, which dynamically acquires images at multiple focal depths for three‐dimensional reconstruction. Because this system relies on contrast gradients rather than light intensity, surface geometry and microtexture can directly affect scan precision. In this study, SHA‐B exhibited the smallest mean linear and angular deviations but the largest variance, suggesting that while its notch and reflective surface enhanced feature recognition, the additional surface irregularity within the notch caused unstable depth reconstruction in repeated scans. This observation aligns with previous findings indicating that local surface irregularities influence registration accuracy depending on the scanner’s imaging mechanism [33]. In contrast, the matte surface of SHA‐I provided insufficient optical contrast for DDS capture, resulting in greater angular deviation. Although both the SHA‐I and the SB were made of nonreflective materials, the SB was taller and had more distinct geometric features, allowing more consistent landmark recognition and smaller angular errors [28, 30]. These findings suggest that, in DDS‐based scanners, both the imaging mechanism and the surface characteristics of the scanned component interactively determine scan accuracy, with moderate reflectivity and well‐defined geometry providing optimal precision.

Although there is no consensus on the clinically acceptable range of linear deviation for prostheses, previous studies have suggested that deviations between 70 and 120 *μ*m are considered acceptable [21, 28]. The results of this study demonstrated statistically significant differences in 3D linear deviations in SHA‐I and SHA‐T at the second premolar and in SHA‐T at the first molar when comparing SB groups with SHA groups for each implant system. However, all SHA groups except for SHAC‐B showed mean 3D linear deviation below 70 *μ*m. These findings suggest that intraoral scanning using SHAs for prosthetic fabrication is clinically acceptable compared with the conventional use of SBs.

In the systematic review conducted by Flügge et al., the mean angular deviation for impressions obtained through the conventional impression technique for partially edentulous jaws was reported as 0.6° (95% CI: 0.5°–0.8°), while the highest deviations in the digital impression technique were reported as 1.6° (95% CI: 1.3°–1.9°) [6]. In the study by Mizumoto et al., average angular deviation ranged from 0.21° to 0.78° depending on the position and scan technique of the SB [27]. Similarly, Batak et al. reported angular deviations of 0.4° to 0.8° depending on the position and height of the SHA [30]. In the present study, mean angular deviations for the SHA groups (excluding the SHAC‐B group) ranged from 0.045° to 0.642°. These results indicate that the angular deviations are either comparable to the previously reported values, demonstrating the potential of SHAs to achieve clinically acceptable accuracy in implant impressions.

Decreased intraoral exposure height of SBs has been associated with increased linear and angular deviations in digital implant impressions [27]. Specifically, Jung et al. [28] reported that limited SB height may hinder effective landmark acquisition, thereby compromising the accuracy of intraoral scanning procedures. Furthermore, the choice of SHAs is often constrained by limited interocclusal space and the need to minimize patient discomfort. These limitations necessitate shorter SHAs in many clinical situations [28, 29]. In this study, the cap used to increase the height of the SHA was applied only to the Bright implant, as it was the only commercially available product for this purpose, and no equivalent caps were available for the other systems. However, adding a plastic cap to the SHA in this study resulted in higher linear and angular deviations. These findings suggest that the increased deviation may result more from the physical properties and manipulation of the cap than from the height alteration alone. Specifically, differences in materials between the SHA (e.g., titanium) and the cap (e.g., PEEK or PEKK), along with the snap‐fit connection mechanism and the slight flexibility of the cap, seem to contribute to an imprecise connection between the SHA and the cap. Thus, the snap‐fit connection appears to prevent accurate mechanical engagement between the SHA and the cap. Therefore, choosing an SHA with a height optimized for clinical needs could maximize its benefits by reducing the need for frequent removal and reinsertion while also minimizing issues related to soft‐tissue adaptation and patient comfort.

A related approach that integrates a healing abutment with a supragingival indexing component has been evaluated in systems that add a scan peg to the healing abutment. In these designs, a key‐and‐keyway design provides precise seating of the peg and offers clearly defined geometric indices for registration, resulting in scan accuracy comparable to that of a SB [14]. In contrast to the elastically retained snap‐on cap assessed in the present study, a tolerance‐controlled keyway interface reduces micromotion and interfacial compliance, indicating that interface precision and material rigidity are crucial when temporarily extending the supragingival height. Accordingly, future height‐extension accessories should incorporate minimal‐clearance keyed couplings and dimensionally stable materials to maintain consistent optical reference points during acquisition while preserving the clinical advantage of avoiding disconnection of the healing abutment.

Beyond mechanical and optical considerations, biological factors may also influence the clinical performance of SHAs. Maintaining the abutment in situ during digital scanning preserves the peri‐epithelial tissue seal and prevents soft‐tissue collapse that can occur with repeated disconnection. Mouhyi et al. demonstrated that the use of coded healing abutments maintained peri‐implant mucosal stability and achieved a high rate of prosthetic precision after 1 year of function [32]. Similarly, Ramadan et al. found that digital impressions obtained using SHAs produced comparable contact accuracy to SBs while significantly reducing chairside time [31]. These studies suggest that SHA‐based workflows not only simplify clinical procedures but may also promote peri‐implant tissue stability by minimizing manipulation of the healing abutment.

The present findings are also in line with previously published studies evaluating digital impression accuracy. Mizumoto and Yilmaz and Batak et al. emphasized that SB geometry and surface characteristics are key determinants of trueness [11, 30], whereas Jung et al. reported that SHAs can achieve accuracy comparable to that of SBs [28]. Revilla‐León et al. further demonstrated that registration accuracy varies depending on the scanner’s optical acquisition mechanism [33]. Collectively, these results support the clinical validity of SHA‐based digital workflows while underscoring the importance of optimizing surface design and imaging technology for improved precision.

### 4.1. This Study Has Several Limitations

As an in vitro study, it did not account for clinical conditions such as patient movement, saliva, or gingival tissue interference, and accuracy was evaluated at the fixture level rather than the prosthesis level, which may not fully reflect the clinical outcomes. Also, variations in scanner types or scanning techniques could potentially alter the results. In addition, accuracy was assessed as absolute 3D linear and angular deviations at individual implant sites after registration to a reference model. Relationship‐based spatial metrics, such as interimplant center‐to‐center distances, axis‐to‐axis inclinations, and tooth‐anchored measurements based on fixed anatomical landmarks, were not analyzed. Further studies are needed to evaluate these spatial relationships and to determine whether group differences influence them.

## 5. Conclusions

Based on the results of this in vitro study, the following conclusions can be derived:

1. The shape of SHAs influences the accuracy of implant impressions; however, the observed deviations generally remain within clinically acceptable limits for prosthesis fabrication.
2. Adding a cap to the SHA decreases impression accuracy, indicating the necessity for further optimization of cap design and connection

## Author Contributions

The authors Han Na Lee and Yeon‐Kyung Park contributed equally to this article, and they were co‐first authors.

## Funding

This study was supported by the Korea Medical Device Development Fund grant funded by the Korea government (the Ministry of Science and ICT, the Ministry of Trade, Industry, and Energy, the Ministry of Health and Welfare, the Ministry of Food and Drug Safety), RS‐2020‐KD000305.

## Conflicts of Interest

The authors declare no conflicts of interest.

## Data Availability

The data that support the findings of this study are available from the corresponding author upon reasonable request.
